# Comprehensive Effects of Photobiomodulation Therapy as an Adjunct to Post-orthodontic Treatment Care: A Systematic Review

**DOI:** 10.3290/j.ohpd.b1075107

**Published:** 2021-03-17

**Authors:** Zhiyi Shan, Ka Wai Frank Wong, Colman McGrath, Min Gu, Yanqi Yang

**Affiliations:** a PhD Candidate, Department of Orthodontics, Faculty of Dentistry, The University of Hong Kong, Hong Kong, SAR, China. First literature reviewer and wrote the manuscript; b PhD Candidate, Department of Orthodontics, Faculty of Dentistry, The University of Hong Kong, Hong Kong, SAR, China. Second literature reviewer.; c Clinical Professor, Department of Applied Oral Science and Community Dental Care, Faculty of Dentistry, The University of Hong Kong, Hong Kong, SAR, China. Idea, proofread the manuscript.; d Clinical Assistant Professor, Department of Orthodontics, Faculty of Dentistry, The University of Hong Kong, Hong Kong, SAR, China. Proofread the manuscript.; e Clinical Associate Professor, Department of Orthodontics, Faculty of Dentistry, The University of Hong Kong, Hong Kong, SAR, China. Responsible for communicating with the other authors about progress, submissions of revisions, final approval of manuscript.

**Keywords:** orthodontic retention, orthodontically induced inflammatory root resorption, photobiomodulation therapy, systematic review

## Abstract

**Purpose:**

To evaluate the comprehensive effects of photobiomodulation (PBM) therapy on teeth after active orthodontic treatment.

**Materials and Methods:**

This systematic review was conducted according to the PRISMA guidelines. Six databases were electronically searched and screened for eligible human and animal studies published up to August 2020. The risk of bias was assessed based on the Cochrane Handbook for Systematic Reviews of Interventions and Systematic Review Centre for Laboratory Experiment Tool. Two independent reviewers performed all procedures in duplicate. Any disagreement was resolved by discussion or consultation with a third reviewer.

**Results:**

A total of 395 records were identified from the initial search up to August 2020. Following screening, 16 full-text articles were reviewed for eligibility (κ > 0.90), and ultimately 9 studies (3 clinical studies and 6 animal studies) were included in this review. The key outcomes observed were ‘tooth position maintenance’ and ‘root resorption rehabilitation’. Two controlled clinical trials and two animal studies supported the preventive effects of PBM therapy on the relapse of post-orthodontic tooth positions, while the other two animal studies reported opposing findings. Regarding root resorption, all evidence supported the rehabilitation potential using PBM therapy for teeth that had undergone orthodontic tooth movement. There was a high risk of bias among studies, except for one randomised controlled trial. Due to the substantial heterogeneity among studies in terms of their types, participants, designs, PBM therapy settings and variables of interest, it was not possible to conduct a meta-analysis; therefore, a qualitative synthesis is presented.

**Conclusion:**

The quality of evidence for PBM therapy contributing to the maintenance of tooth position or improved dental health after orthodontic treatment remains low. There is considerable controversy over the effects of PBM therapy on orthodontic relapse. However, the use of PBM therapy after orthodontic treatment has promising effects for root resorption rehabilitation and is generally recommended.

The stability of satisfactory results achieved by orthodontic treatment is of great importance for both patients and clinicians, whose expectations regarding outcomes are high. Harmonious occlusion and some degree of over-correction is recommended at the finishing stage. Long-term administration of retainers (e.g. Hawley’s), lingual-fixed appliances or vacuum-formed retainers, are widely accepted as clinical routine to maintain the results acquired by orthodontic tooth movement (OTM). Despite these conventional regimens, orthodontic relapse, defined as immediate or postponed drifting of teeth towards their original sites, is still inevitable in clinical practice.^46,67^ This is especially true for teeth with initial rotation or with compromised periodontal support. Strategies have been proposed to supplement the conventional retention regimen, including circumferential supracrestal fiberotomy,^21^ local injection of biological reagents^2,31,34^ and prescription of some cardiovascular medications.^17,47^ However, these approaches have met with limited success and acceptance, due to their drawbacks such as the introduction of invasive wounds, uncertain efficacy for the patients, and potential systemic side effects.^6^

One main reason for the difficulty in preventing orthodontic relapse may be the cellular and molecular changes underlying orthodontically repositioned teeth. Previous views on relapse suggest that it is caused by the release of mechanical force stored in deformed collagen fibers in supra-alveolar areas during OTM.^8,22,60^ In the late 1990s, Redlich et al^55^ established that the changes in the elastic properties of gingival tissues are the main cause of relapse, rather than collagen fibers. Several subsequent studies also highlighted the role of hard tissues in post-orthodontic instability and suggested that the remodelling of all surrounding tissues contributes to orthodontic relapse. In terms of soft tissues, collagen fibers have been shown to influence short-term relapse, whereas elastic fibers contribute more to long-term mobility due to their extensive mutual crosslinks and slower rate of degradation.^32,48^ Regarding hard tissues, researchers found that adjacent alveolar bone undergoes a similar procedure between relapse and active treatment^18,25,26^ when osteogenesis and osteoclastogenesis both significantly participate in tooth resettlement. Accordingly, manipulating the metabolism of adjacent hard and soft tissues appears to be a promising approach to alleviate or even prevent orthodontic relapse.

Photobiomodulation (PBM) therapy is an adjunctive, non-invasive, highly compatible medical treatment for multiple indications in oral medicine, including aphthous stomatitis, periodontal disease, dental hypersensitivity, and orofacial pain.^15, 6,30,52,53,56,59,63^ The name describes its mechanism of regulating biological metabolic activities with photons.^19^ Fibroblasts from gingival and periodontal ligament were reported to upregulate their anabolic activities and expression of mediators, such as heat shock proteins, transforming growth factor β, β-defensin 2, and basic fibroblast growth factor after PBM irradiation.^1,6,57^ On the histological level, PBM-irradiated teeth showed a significant reduction in the coverage of non-epithelium gingival surfaces with less inflammation after gingivectomy.^43^ With regard to hard tissues, osteoblastic-like cells are also reported to be susceptible to PBM therapy and to increase their potential for proliferation, adhesion, differentiation and mineralisation at specific settings.^5,14,42,66^ Several clinical studies have documented that PBM therapy could enhance the stability of bone-anchored mini-screws during active loading^23,28, 51,52,71^ and also accelerate bone regeneration at enlarged midpalatal sutures while expanding the maxillary width;^24,29,64^ these effects can be attributed to a higher mineral apposition rate in laser-irradiated areas, as revealed by radiographic examination. Therefore, it appears that in principle, PBM therapy could be a promising adjunct to the conventional retention regimen and compensate for post-OTM risks by modulating bone and soft tissue metabolism.

Recently, the application of PBM therapy in the orthodontic field has been proven effective in OTM acceleration and pain alleviation,^18,20,53,58,59^ but it remains inconclusive as to whether PBM therapy impacts post-OTM tooth status. One previous study surveyed clinical trials investigating the effects of PBM on orthodontic relapse, but with limited subject numbers and no eligible studies.^65^ Another study synthesised the evidence from both human and animal studies, but only considered the effects on rotational relapse instead of all tooth conditions after active orthodontic treatment.^49^ This systematic review was conducted to analyse current evidence on the effects of PBM on teeth which have undergone OTM. The study questions were: 1. Is there adequate evidence that PBM therapy helps to maintain tooth position during the post-OTM period? 2. Is there adequate evidence that PBM therapy improves dental health during the post-OTM period?

## Materials and Methods

This systematic review was performed and reported following the instructions of the Preferred Reporting Items for Systematic Reviews and Meta-Analyses (PRISMA) guidelines.^45,50^ The protocol was prospectively registered on the PROSPERO online database (CRD49019132133).

### Search Strategy

Two reviewers (ZYS and FW) independently conducted a systematic electronic search of six major databases, namely Cochrane Central Register of Controlled Trials, MEDLINE (via Ovid 1946), Embase (via Ovid 1974), Pubmed (1997), Scopus and ProQuest, for articles published up to August 2020. Clinicaltrails.gov was also included to avoid omissions of ongoing clinical studies. Medical subject headings, free text words and their synonyms were applied as search terms, including ‘orthodontic/appliance/force’, ‘retention/maintenance/stability/relapse’ and ‘low-level laser/low-intensity laser/soft laser/photomodulation’. The detailed search strategy is presented in the Appendix.

### Study Selection

The eligibility criteria are listed in Table 1. Accordingly, all titles and abstracts obtained from the electronic search were independently screened by the two reviewers ZYS and FW. Full articles were retrieved for final assessment and their reference lists were also screened based on the aforementioned criteria. During the process, any disagreement between the two reviewers was resolved by discussion or consultation with a highly experienced reviewer (YQY). Cohen’s Kappa values were computed to verify the inter-reviewer reliability, which was considered acceptable if not lower than 0.6.

**Table 1:** Eligibility criteria in PICOS format

	Population	Intervention	Comparators	Outcomes	Study
Inclusion criteria	Teeth of patients who had undergone LLLT after active OTM Teeth of experimental animals with low-level laser irradiation following the termination of an active orthodontic stage Systemically health subjects	Low-level laser irradiation during the post-orthodontic stage following active OTM Post-orthodontic management with or without retainers	Negative control: Teeth without OTM and LLLT Positive control: Teeth had gone through active OTM but without LLLT Others: Teeth irradiated with LLLT before or during OTM	Primary outcomes: Post-OTM tooth movement, dental health Secondary outcomes: Histological or biochemical changes	Clinical studies including randomised-controlled and non-randomised controlled trials Experimental animal studies
Exclusion criteria	Subjects with severe maxillofacial deformities or who had undergone any orthopaedic or surgical procedures Subjects was pregnant, lactating, ovariectomised, or under any pharmaceutical medications	Delivery of LLLT before or during the process of active tooth movement	None	Studies without demonstrating a single primary outcome listed above	In vitro studies, case reports, reviews, personal opinions and technique description articles without sample reporting

### Data Extraction and Analysis

The following data were extracted: general information (first author and year of publication); study type and design; participants and target teeth (sample size and characteristics); orthodontic regimen (active and post-active orthodontic strategy and period); photobiomodulation protocols (types, wavelength, beam size, mode, output power, dosage density, time of onset, duration, frequency and method of delivery); assessments (approach, region of interest, outcome variables and time-points); primary outcomes (post-OTM tooth movement or related dental health); and secondary outcomes related to histological or biochemical changes.

### Risk of Bias Assessment

The assessment for the risk of bias of all human studies was performed in RevMan5.3^61^ using the Cochrane Risk of Bias Tool.^33^ Seven domains were considered: (1) random sequence generation; (2) allocation concealment; (3) blinding of participants and personnel; (4) blinding of outcome assessment; (5) incomplete outcome data; (6) selective reporting; and (7) other bias. For animal studies, the risk of bias was assessed based on the Systematic Review Centre for Laboratory Experiment Tool (SYRCLE tool).^35^ Ten domains covering bias from subject selection, intervention performance, outcome detection, attrition, and reporting were considered for grading the quality of evidence.

### Data Synthesis

The data of interest from human and animal studies were synthesised separately because of their substantial difference in nature. Within each study type, data extracted on the aforementioned aspects were further assessed for heterogeneity. If both clinical and statistical homogeneity were achievable, quantitative synthesis and meta-analysis of the retrieved data would be performed; otherwise, a narrative description would be presented.

## Results

### Characteristics of Selected Studies

**Fig 1:**
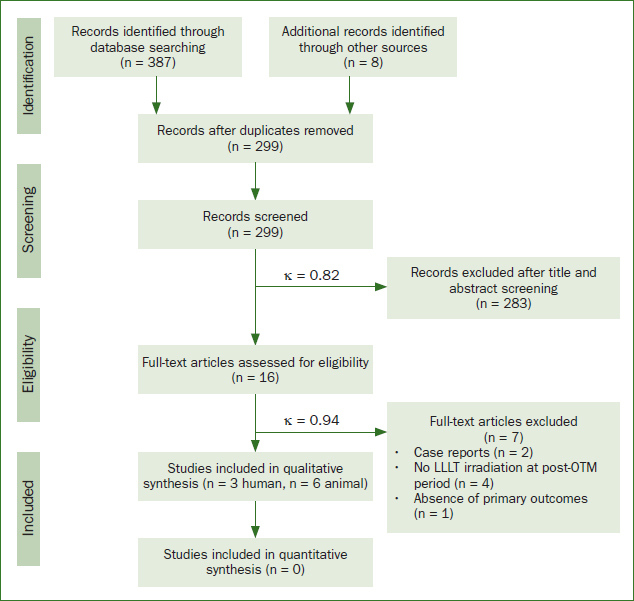
PRISMA flow diagram summarising the literature search.

The process of study search and selection is illustrated in Fig 1. The electronic search up to August 2020 yielded a total of 387 relevant records from the six databases; another eight records were identified by screening bibliographies. After removing duplicates, the remaining 299 studies were analysed by title and abstract, which left 16 articles for full-text evaluation. According to the eligibility criteria, seven of these articles were further excluded for different reasons. Finally, this systematic review included three clinical trials^38,39,73^ and six animal studies.^3,14,27,40,44,54^ The final Cohen’s kappa coefficient value was 0.94 for full-text selection, indicating a perfect agreement between the two reviewers.

Five studies were focused on tooth position maintenance after active orthodontic treatment: two were controlled clinical trials (CCTs),^38,73^ and the others were animal experiments.^27,40,44^ Two types of tooth movement were covered, including rotational and transitional relapse. Three other studies – one RCT^38^ and two animal studies^3,14^ – examined orthodontically induced inflammatory root resorption (OIIRR), which was one common post-OTM condition compromising dental health. One individual animal study contained information relating to both of the above outcomes.^54^ No studies shed light on other dental problems after orthodontic treatment, and no harmful effects of PBM therapy were ever reported. Considering the limited number of studies for each outcome and their considerable heterogeneity in terms of their types, participants, designs, PBM settings, and variables of interest (Table 2), no meta-analysis could be performed. Therefore, a qualitative synthesis of PBM effects on post-OTM teeth was performed in a narrative manner.

**Table 2:** Detailed low-level laser regimens for all included studies

Study types	Outcome	Authors and year [Ref]	Laser type	Wavelength (nm)	Beam size (cm^2^)	Power (mW)	Mode	Dosage density (J/cm^2^)	Energy (J)	Timing of first irradiation	Frequency and times	Irradiation period per session (s)	Method of irradiation
Clinical studies	OR	Zahra et al (2009) [73]	GaAs	904	0.5	30	Pulsed (9999 Hz)	4.9	16.2	Within the first week after diastema closed but before debonding	Every second day (3 sessions)	180	Contact, covering an area of 3.3 cm^2^
		Jahanbin et al (2014) [38]	GaAlAs	810	0.28	200	Continuous	35.7	80	At the finishing stage of orthodontic treatment	Twice a week (8 sessions)	200	Contact, 4 points
	OIIRR	Khaw et al (2017) [39]	AlGaInP	660	0.26	75	Continuous	3.6	45	4 weeks after active orthodontic force	Once a week (6 sessions)	120	Contact, 8 points
Animal studies	OR	Kim et al (2010) [40]	GaAlAs	808	NR	763;	Pulsed (10 Hz)	4.63-6.47	NR	Immediately after orthodontic couple force removed	Every 3 days (9 sessions)	240	2–3 mm from the gingiva, 8 points
		Franzen et al (2015) [27]	GaAlAs	830	0.13	75	Continuous	23	3-21	Immediately after appliance removal	Evenly distributed during relapse period (1, 2, 3, 4, 5, 7 sessions)	17	Contact from the occlusal and lingual sides
		Lee et al (2016) [44]	GaAlAs	780	NR	NR	Continuous	20	NR	After 1-week temporal retention	Daily until sacrificed (2, 4, 6 sessions)	NR	Contact, over the root areas
	OIIRR	Altan et al (2015) [3]	GaAlAs	820	0.208	50	Continuous	4.8	4.2	After 11-day OTM and removal of force appliance	Every other day for 2 weeks (7 sessions)	12	Contact, 4 points
		Conti et al (2019) [14]	GaAlAs	810	0.02	100	Continuous	75	12	After 7-day OTM and removal of force apparatus	Day 7, 9, 11, and 13 (4 sessions)	30	Contact, 2 points
		Ozturk et al (2020) [54]	GaAlAs	SW: 650; CW: 532-650-940	100	100	Continuous	18	18	One day before force appliance removal	9 times with a 1-day interval	180	Contact, over the root areas

OR: orthodontic retention/relapse; OIIRR: orthodontically-induced inflammatory root resorption; GaAs: gallium-arsenide; GaAlAs: gallium-aluminum-arsenide; AlGaInP: aluminum- gallium-indium-phosphide; SW: single wavelength; CW: cumulative wavelength; NR: not reported.

### Effects on Post-OTM Tooth Position Maintenance

Two CCTs^38,73^ and four animal studies.^27,40,44,54^ fulfilled the inclusion criteria investigating the effects of PBM therapy on tooth position maintenance after OTM. Detailed information is summarised in Table 3. The favourable preventive effects of PBM on tooth retention were reported in both CCTs.^38,73^ One study showed that teeth irradiated with a low-level laser (GaAlAs, 810 nm, continuous wave, 35.7 J/cm^2^) had a nearly 60% decrease in the degree of post-OTM relapse compared with their control counterparts (p < 0.05).^38^ The other study using GaAs laser (904 nm, pulse wave, 4.9 J/cm^2^) detected a statistically non-significant reduction in orthodontic relapse, with the exception of a statistically significant increase in the alveolar crest height and a substantial slowing of bone density reduction in irradiated areas.^73^

**Table 3:** Characteristics of included studies on tooth position

				Intervention			Assessment				
Study type	Authors and year [Ref]	Study model/design	Participants and teeth	Active force and period	Adjunctive post-OTM strategy	Observe period	Measure method	Target regions	Outcome variables	Comparators	Outcomes
Clinical studies	Zahra et al (2009) [73]	CCT, parallel	N = 14 (Nm = 3, Nf = 11) age 19–27 years	FA; 8-16 months	45 days FA; 6 months Hawley retainer	15 days, 45 days, 3 monts, 6 monts, 1.5 years	Direct measurement on study model	Incisal region and adjacent alveolar bones	Diastema size Changes of alveolar bone density and height	G1: LLLT (n = 7) G2: control (n = 7)	Diastema reopening less in G1, but without statistically significant difference. The growth of alveolar bone height is statistically significantly higher in G1 at 3 months, 6 months and 1.5 years; (p < 0.05). Bone density less reduced in G1 at 6 months.
	Jahanbin et al (2014) [38]	CCT, parallel	N = 24 (Nm = 4, Nf = 20) age 16-32 years; n = 47	FA; N/A	No retention	1 month	Computer measurements on std. photos	Pretreatment-rotated incisors	Percentage relapse	G1: CSF (N = 6, n = 13) G2: laser-aided CSF (N = 6, n = 11); G3: LLLT (N = 6, n = 12); G4: control (N = 6, n=11)	Percentage of relapse occurred: CSF group (9.66%); laser-aided CSF (12.71%); LLLT group (11.67%); control group (27.82%). Relapse was statistically significantly greater in the control group than the other experimental groups (p < 0.05).
Animal studies	Kim et al (2010) [40]	Male dogs parallel	N=9, n=18	Rotational couple force 50 g; 4 weeks	No retention	4 weeks	Computer measurements on std. photos of study model	Mandibular lateral incisors	Amount of relapse, sulcus depth, gingival recession, connective tissue rearrangement	G1: laser-aided CSF (n = 6) G2: LLLT (n = 6) G3: control (n = 6)	Means percentage of relapse: G1 (14.52±3.59%); G2 (56.80±10.98%); G3 (41.29±5.65%); (p < 0.001). No statistically significant differences in sulcus depth, gingival recession, histologic findings.
	Franzen et al (2015) [27]	Male rats parallel	N = 61, n = 61	50 g closing force; 10 days	No retention	Day 1, 3, 5, 7, 14, and 21	Direct measurement using feeler gauge Densitometric analysis, histological analysis	Maxillary right first molars	Relapse percentage and rate, osteoclasts cell number, bone density	G1: control (n = 35); G2: LLLT (n = 26)	Mean relapse percentage no statistically significant difference. 1 day: G1(62.5%) and G2(54.17%) 21 days: G1(86.11%) and G2(72.22%). Increased numbers of osteoclasts in nearly all experimental groups compared with non-irradiated molars although not statistically significant. Bone density no statistically significant difference.
	Lee et al (2016) [44]	Male rats parallel	N = 52, n = 52	Space-open force; 14 days	1-week retention	Day 8, 10 and 13	Measurement on study model, real-time RT-PCR, immuno-histochemistry analysis	Maxillary central incisors	Relapse rate; relative mRNA translation and protein expression (MMPs)	G1: positive control (n = 12); G2: LLLT (n = 12); G3: doxycycline (n = 12); G4: LLLT + doxycycline (n = 12); G5: negative control (n = 4);	Relapse rate: G1 < G2 (p < 0.05). 1 day: G1 (20.70±6.3%) and G2 (27.90±9.7%) 3 days: G1 (23.30±5.1%) and G2 (33.40±8.4%); 5 days: G1 (33.10±7.5%) and G2 (52.00±7.0%). G2 has greatest recruitment of osteoclast-like cells as well as a greater ratio of immunoreactive cells for all tested MMPs.
	Ozturk et al (2020) [54]	Female rats, parallel	N = 33, n = 66	50 g closing force, 10 days	With and without capping composite resin for tooth retention	15 days	3D digital model	First maxillary molars	Active and retention tooth movement	G1: negative control (n = 10) G2: OTM (n = 10) G3: OTM + retainer (n = 10) G4: OTM + retainer + SW-PBM (n = 18) G5: OTM + retainer + CW-PBM (n = 18)	Retention tooth movement (RTM): G1 (0.124±0.020); G2 (1.376±0.072); G3 (0.213±0.182); G4 (0.207±0.090); G5 (0.190±0.079); no statistically significant difference between G3 vs G4, and G3 vs G5. A statistically significant difference was observed in the level of RANKL and COX-2 for G4 and G5 in comparison with G2 and G3 (p < 0.05), but no statistically significant changes in OPG among all groups.

CCT: clinical controlled trial; SMD: split-mouth design; N: number of participants; n: number of evaluated teeth; FA: fixed appliances; CSF: circumferential supracrestal fiberotomy; MMP: matrix metalloproteinase; LLLT: low-level laser therapy.

Although the two studies consistently reported PBM’s positive effects on tooth position maintenance after active orthodontic treatment, their methodologies differed in several ways. For instance, two types of tooth movement were discussed; one study observed transitional tooth movement,^73^ whereas the other study examined the impacts on rotational relapse.^38^ Furthermore, the two studies adopted different retention regimens. One left the fixed appliances attached for the first 45 days and then used Hawley’s retainers for the following 6 months,^73^ whereas the other did not use any post-OTM retainers.^38^ In addition, the adopted PBM parameters were quite different in terms of wavelength, wave mode, and dosage density. Finally, the assessment timepoints were also not comparable: one study conducted long-term observation (1.5 years),^73^ while the other had an observation period of 30 days post-OTM.^38^

The animal studies investigating the effects of PBM therapy on post-orthodontic tooth movement include one using a canine model that assessed rotational relapse^40^ and three on transitional movement with rodent models.^27,44,54^ In terms of rotational relapse, Kim et al^41^ found a significant increase (p < 0.05) in post-treatment tooth stability of approximately 15% when no retainers were applied to GaAlAs laser- (808 nm, pulse wave, 4.63–6.47 J/cm^2^) irradiated teeth compared to their counterparts. Concerning transitional relapse, one study^27^ applied GaAlAs laser (830 nm, continuous wave, 23 J/cm^2^) immediately after active tooth movement, and did not use any type of retainers. They found a positive effect of PBM therapy on tooth position maintenance, but this was not statistically significant (p > 0.05).^27^ In contrast, another study^44^ that allowed a period of tooth retention and applied GaAlAs (780 nm, continuous wave, 20 J/cm^2^) one week after force appliance removal found a negative effect of PBM on post-OTM tooth stability (p < 0.05). Recently, another study^54^ investigated the adjunctive effects of GaAlAs laser (650 nm or 572-650-940 nm, continuous wave, 18 J/cm^2^) with retainers on tooth stability, finding a decreased tendency of relapse for teeth irradiated with PBM compared to those without. However, this difference was not statistically significant (p > 0.05). Interestingly, despite diverse post-treatment settings, all rodent studies^27,44,54^ found some cellular or molecular activities, indicating enhanced osteogenesis and decreased osteoclastogenesis with PBM application.

### Effects on Root Resorption Rehabilitation

One RCT^39^ and three animal experiments^3,14,54^ all showed some level of rehabilitative effect of PBM therapy on root surfaces following active OTM. The details of all included studies are summarised in Table 4. The RCT that prescribed PBM (AlGaInP, 660 nm, continuous wave, 3.6 J/cm^2^) applied it to one side of patients’ maxillary first premolars immediately after the removal of a buccal tipping force during retention, whereas their counterparts on the opposite side of the dental arch were subjected to a placebo laser. Six weeks post OTM, the mean total crater volume on the root surfaces of laser-irradiated teeth was 0.033 ± 0.039 mm^3^ less than that of the placebo-irradiated teeth. However, this difference was not statistically significant (p > 0.05).^39^

**Table 4:** Characteristics of included studies on orthodontically-induced inflammatory root resorption

				Intervention		Assessment					
Study type	Authors and year [Ref]	Study model/design	Participant and teeth	Active force and period	Adjunctive post-OTM strategy	Observe period	Measure method	Target regions	Outcome variables	Comparators	Outcomes
Clinical study	Khaw et al (2017) [39]	RCT, SMD	N = 20, n = 40 (Nm = 12, Nf = 8) aged 13–19 years	150 g buccal tipping force; 4 weeks	0.018 SS FA	6 weeks	Micro-CT	Extracted first premolars	Crater volume	G1: lased group (n = 20) G2: sham group (n = 20)	The mean total crater volumes in G1 was 0.033±0.039 mm^3^ less than that of G2, but the difference is not statistically significantly different. No additional side effects detected.
Animal studies	Altan et al (2015) [3]	Male rats, parallel	N = 30, n = 35	50 g closing force; 11 days	Composite resin in the interdental space	14 days	Histochemical evaluation, immunohistochemical evaluation	Left maxillary first molars	Semi-quantitative evaluation (4-degree grade) of OB, FB, capillary, inflammatory cells, RR, RANKL, OPG	G1: OTM (n = 7) G2: OTM + LLLT (n = 7) G3: OTM + 14-day retention (n = 7) G4: OTM + 14-day retention + LLLT (n = 7) G5: control (n = 7)	No statistically significant difference existed in RR between G1 and G2. A statistically significant decrease in the amount of RR for G4 in comparison with G3 (p = 0.02). The number of osteoblasts and fibroblasts notably increased in G2 and G4 compared with G1 and G3 respectively (p < 0.001). RANKL/OPG ratio: G1 > G3 > G2 > G4.
	Conti et al (2019) [14]	Male rats, SMD	N = 20, n = 40	50 g closing force; 7 days	No retention	7 days	Histochemical evaluation, immunohistochemical evaluation	First maxillary molars	Pooled areas of RR lacunae RANKL & OPG	G1: no OTM (n = 10) G2: OTM (n = 10) G3: OTM + 7-day healing (n = 10) G4: OTM + 7-day healing + LLLT (n = 10)	A statistically significant increase in the total area of RR in G1, G2, and G3 when compared to the G1 group on compression side of roots (p < 0.05). In the compression side of the distal root, there was a statistically significant increase in RR area in the G3 compared to the G2 and G4 groups (p < 0.05). G4 showed a statistically significantly higher OPG expression at both compression and tension sides compared to G1-G3. (p < 0.05). G4 showed a statistically significantly less RANKL expression at tension sides compared with G1-3 (p < 0.05).
	Ozturk et al (2020) [54]	Female rats, parallel	N = 33, n = 66	50 g closing force; 10 days	With and without capping composite resin for tooth retention	15 days;	Micro-CT; RT-PCR;	First maxillary molars	Volumetric and linear measurement of RR; RANKL, OPG, COX-2;	G1: negative control, no OTM (n = 10) G2: OTM (n = 10) G3: OTM + retainer (n = 10) G4: OTM + retainer + SW-PBM (n = 18) G5: OTM + retainer + CW-PBM (n = 18)	Resorption lacunae volume (p < 0.001), number of resorption lacunae (p < 0.05), and percentage of the resorption (PR) lacunae (p < 0.001) decreased with PBM applications when compared with the positive control groups, and the mean PR was similar in G1 when compared G4 (p > 0.05). PBM applications showed marked inhibitory and reparative effects on OIIRR by modulating the RANKL and COX-2 expression levels (p < 0.05), but no statistically significant changes in OPG (p > 0.05).

RCT: randomised controlled trial; SMD: split-mouth design; N: number of participants; n: number of evaluated teeth; FA: fixed appliances; OTM: orthodontic tooth movement; OB: osteoblasts; OC: osteoclasts; RR: root resorption; RANKL: receptor activator of nuclear factor kappa-B ligand; OPG: osteoprotegerin; COX-2: cyclooxygenase-2.

The three experimental animal studies^3,14,54^ all found a statistically significant difference in the OIIRR for teeth irradiated with PBM post-treatment compared to their counterparts (p < 0.05). In addition, they also discovered consistent cellular or molecular activities favouring bone or root surface reconstruction. However, some methodological variations existed, which required caution during data synthesis. First, teeth were differently managed after active orthodontic treatment; two of the studies performed irradiation concurrent with tooth retention,^3,54^ while the other did not use retainers.^14^ Second, there was a considerable difference in the PBM therapy parameters: one used laser with 4.8 J/cm^2^ (820 nm, continuous wave),^3^ another applied a much higher dosage density (810 nm, continuous wave, 75 J/cm^2^),^14^ while the third study employed a moderate dosage density (continuous wave, 18 J/cm^2^) and two light configurations (single wavelength of 650 nm and cumulative wavelengths of 532-650-940 nm).^54^ Finally, the approaches and outcomes for assessing OIIRR varied. Two of the studies were based on histochemical and immunohistochemical analysis,^3,14^ while the other employed micro-CT for both volumetric and linear evaluation.^54^

### Quality Evaluation

**Fig 2:**
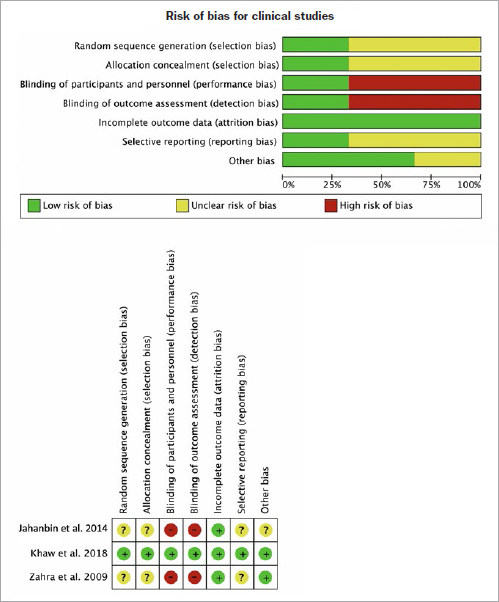
Assessment of risk of bias item across all included clinical studies.

The quality of the three included clinical studies was assessed according to the guidelines of the Cochrane Risk of Bias Tool. The two CCTs^38,73^ were ranked as having a high risk of bias, with emphasis on the lack of blinding and randomisation. Additionally, confounders existed during post-OTM PBM application, including shifting the retention regimen or a possible crossover effect due to light scattering.^38^ In contrast, the evidence provided by the RCT was of high quality, as risk of bias in all seven domains was rated as low^39^ (Fig 2).

**Fig 3:**
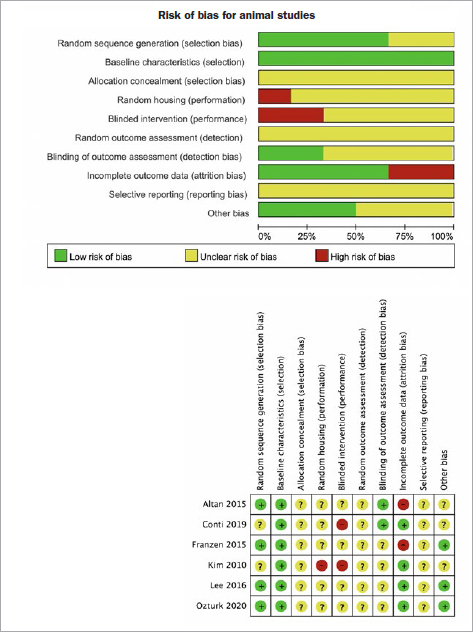
Assessment of risk of bias item across all included animal studies.

The quality of animal studies was assessed using the Systematic Review of Experimental Animal Studies (SYRCLE) risk of bias tool. Four out of six studies presented high risks of bias in at least one domain and were therefore rated as having low quality of evidence.^3,16,27,40^ The other two studies did not specify their handling of allocation concealment, random housing of the animals and outcome assessment, blinded intervention and outcome assessment, and selective reporting.^44,54^ Figure 3 schematically presents the results of risk of bias in animal studies.

## Discussion

Of all the included studies evaluating the effects of PBM therapy on post-OTM tooth stability, two discussed rotational relapse.^38,40^ In clinical practice, orthodontically de-rotated teeth are more likely to return to their original state, even when orthodontic retainers are routinely administered. Past studies have revealed that soft tissue turnover, i.e. the remodelling of collagen and elastic fibers, plays a vital role in the occurrence of rotational relapse.^11^ Based on this, some researchers hypothesised that the biomodulation of soft tissues by PBM therapy might be a promising approach to prevent post-OTM rotation. However, according to the results of the two relevant studies, the impacts of PBM could be either positive or negative, depending on various factors. In line with the biphasic dosage-response theory,^37^ the first factor is the dosage density. Jahanbin et al^38^ found that a GaAlAs laser with 810-nm wavelength could alleviate the degree of rotational relapse when the dosage density was high at 35.7 J/cm^2^. In contrast, Kim et al^40^ used the same type of laser (GaAlAs, 808 nm) with a low dosage density (4.63–6.47 J/cm^2^) and found that it decreased post-treatment tooth stability. One systematic review of in vitro studies reported that laser with a dosage density < 16 J/cm^2^ could promote fibroblast growth, proliferation and osteogenic differentiation, whereas laser with an extremely high dosage density exhibited inhibitory effects.^57^ It is possible that the effects of PBM therapy on rotational relapse also follow the same rules, converting fibroblasts from predominantly anabolic to catabolic activities, corresponding to a shift from adverse effects to positive ones along with the increase in dosage density.^41^ However, this interpretation only applies to teeth free of movement after the immediate termination of active forces. As Kim et al^40^ suggests, PBM therapy could act differently on the orthodontic outcomes for teeth with and without retainers by stimulating soft tissue metabolism. Therefore, whether the adjunctive PBM enhances the efficacy of conventional retention appliances is still unclear. Finally, there are other confounders that prevent any generalisation of the effects of PBM therapy on rotational relapse, including substantial heterogeneity in the characteristics of subjects and the initial status of the experimental teeth. In addition, both articles have a high risk of bias because of a limited number of subjects and no sample size calculations. Further investigations with a higher quality of evidence are thus warranted.

The other two post-OTM outcomes discussed by the remaining seven studies, i.e. transitional relapse and root resorption, are both closely related to the activities of osteoblast-like cells and osteoclast-like cells for hard tissue remodelling. On the one hand, after the termination of active forces, alveolar processes generate some hyalinised areas in response to the released mechanical forces, which then trigger osteoclast recruitment and bone resorption in the direction of tooth relapse on the previous tension side. Meanwhile, more anabolic activities such as osteoblast proliferation and differentiation occur on the opposite side, leading to bone regeneration against the direction of tooth relapse to compensate for previous bone resorption.^25,26,72^ On the other hand, pathological OIIRR occurs during OTM when osteoclastic-like cells accumulate near the root surfaces.^9,10^ After termination of orthodontic force, physiological repair would follow involving the deposition of uncalcified-cementoid matrix, fibroblast-like cells, and cementoblast cells as well as the detachment of clastic cells.^12^ Past cellular^13^ and molecular investigations^36^ have documented the capacity of PBM to modulate the activities of osteoblasts and osteoclasts with bone-related biomarkers such as RANKL and OPG. This provides a biological justification for applying PBM to prevent transitional relapse and OIIRR. However, this theory has yet to be supported by in situ studies.

Among the four studies on transitional relapse,^27,44,54,73^ three failed to observe any statistically significant reduction in the amount of post-OTM displacement for PBM-irradiated teeth after the termination of active forces.^27,54,73^ In contrast, one study showed a statistically significantly detrimental effect of PBM therapy on tooth position maintenance.^54^ It is true that the diversity of laser types and parameter settings might be one reason for this inconsistency. However, because a general susceptibility of osteoblast-like cells to multiple laser parameters without a clear specificity has been reported,^13^ the above discrepancy might be attributable to variations in the retention regimen; two studies implemented PBM therapy immediately after OTM,^27,73^ whereas the other studies delivered irradiation during^54^ or after^44^ a period of tooth retention. The lag of adjacent alveolar reconstruction is the primary reason for transitional relapse; therefore, the effects of PBM therapy on tooth position maintenance might skip the critical period and recede with time.

In comparison, the results of studies on OIIRR are more consistent^3,14,39,54^ and in line with previous findings on teeth without OTM, suggesting that PBM therapy could enhance the development of roots and stimulate the proliferation of cementoblasts, which contribute to secondary cementum formation.^4,68^ All three animal studies^3,14,54^ included in this systematic review reported statistically significantly favourable effects of PBM therapy during the post-orthodontic period, and the RCT^39^ showed a generally decreased tendency toward OIIRR for teeth irradiated with PBM compared to their counterparts, but this was not statistically significant. The latter statistically non-significant difference was not surprising,^39^ since there was a much longer observation period (6 weeks), lower irradiation frequency (once a week), and parameter differences in comparison with other animal studies.

It is clear that current evidence is insufficient to deduce the effects of PBM therapy on the prognosis of orthodontic treatment after the active OTM stage, in terms of outcomes for both tooth relapse and OIIRR. One barrier to generalising these results is the afore-mentioned methodological discrepancies; another is the fact that the underlying cellular and molecular mechanisms are not yet fully understood. A classical theory for the PBM effect considers the activities of cytochrome C oxidase (CCO) in the respiratory chain, which are boosted by photons in the red and infrared wavelengths that penetrate the mitochondria.^70^ By greatly enhancing adenosine triphosphate (ATP) production and vital second messengers, such as nitric oxide and reactive oxygen species, PBM regulates various metabolic activities such as cell proliferation, migration, adhesion and apoptosis. However, this theory cannot explain the inconsistencies between some therapeutic laser wavelengths and the absorption spectra of CCO. Another hypothesis, the ‘water oscillator paradox’, was proposed by Santana-Blank et al,^62^ implicating that intracellular water dynamics also play an essential role in PBM effects. Recently, Wang et al^69^ found that heat/light-gated ion channels seem to be the primary photoreceptor for 980-nm wavelength lasers, whereas CCO is the primary photoreceptor for the 810-nm wavelength. Most studies reviewed here used PBM in the 808–830-nm wavelength range,^3,14,27,38,40^ except for two studies that used a 650–660-nm laser for OIIRR^39,54^ and two that used 780-nm^44^ and 904-nm^73^ lasers for transitional relapse. Considering that chromophores might alternate with different wavelengths, the optimal dosage for achieving favourable PBM effects can vary and may significantly influence post-OTM tooth status.

This systematic review is the first to comprehensively evaluate the effects of PBM therapy on teeth in post-OTM scenarios, aiming to justify its application for plausible orthodontic prognosis. The present study showed that considerable controversy exists on the effects of PBM therapy on post-OTM tooth stability, but its use for rehabilitation effects on root resorption are generally recommended. However, great heterogeneity was noted among study subjects, types, PBM parameters, post-OTM strategies, and assessment methods. Moreover, most studies suffered from limitations including small sample sizes, high risk of bias, relatively short observation periods, and a paucity of demonstrations of cellular and molecular mechanism. Therefore, more well-designed studies with broader PBM parameters and more consistent orthodontic and post-OTM settings are needed in the near future.

## Conclusion

The quality of evidence that PBM therapy contributes to the maintenance of tooth position or improved dental health after orthodontic treatment remains low. There is considerable controversy over the effects of PBM therapy on orthodontic relapse. However, the use of PBM therapy after OTM has promising effects for root resorption rehabilitation and is generally recommended.

## Appendix search strategy

**Table:**

Database	Search strategy
CENTRAL (The cochrane library)	#1 MeSH descriptor: [Orthodontics] explode all trees #2 malocclusion #3 appliance.tw. or orthodontic* force.tw. or active force.tw #4 MeSH descriptor: [Laser Therapy] explode all trees #5 light therapy.tw. or photobiomodulation.tw. or laser irradiation.tw. or phototherap*.tw. or diode laser.tw. or low-level laser.tw. or low-intensity laser.tw. or low-power laser.tw. or soft laser.tw. or therapeutic laser.tw. #6 postorthodontic OR post-orthodontic* OR post orthodontic* #7 MeSH descriptor: [Recurrence] explode all trees #8 remov* AND orthodontic* #9 relaps* OR reten* OR recur* OR stability OR retain* OR maintenance #10 #4 OR #5 #11 #6 OR #7 OR #8 OR #9 #12 #1 OR #2 OR #3 #13 #10 AND #11 AND #12
Medline & MEDLINE(R) In-Process & Other Non-Indexed Citations	#1 exp orthodontics/ #2 malocclusion/ #3 (appliance or orthodontic force or active force).tw. #4 exp low level laser therapy/ #5 light therapy or laser irradiation or phototherapy or photobiomodulation or diode laser or low power laser or low intensity laser or soft laser or therapeutic laser).tw. #6 exp relapse/ or exp recurrence/ #7 (post ortho* or post-ortho* or remov* force).tw. #8 (relap* or reten* or stab* or retain* or reopen or mainten*).tw #9 #1 or #2 or #3 #10 #4 or#5 #11 #6 or #7 or #8 #12 #9 and #10 and #11
EMBASE	#1 exp orthodontics/ #2 malocclusion/ #3 (appliance or orthodontic force or active force).tw. #4 exp low level laser therapy/ #5 light therapy or laser irradiation or phototherapy or photobiomodulation or diode laser or low power laser or low intensity laser or soft laser or therapeutic laser).tw. #6 exp relapse/ or exp recurrence/ #7 (post ortho* or post-ortho* or remov* force).tw. #8 (relap* or reten* or stab* or retain* or reopen or mainten*).tw #9 #1 or #2 or #3 #10 #4 or#5 #11 #6 or #7 or #8 #12 #9 and #10 and #11
Scopus	#1 TITLE-ABS-KEY (orthodontic* OR appliance OR “orthodontic force” OR “active force”) #2 TITLE-ABS-KEY (“laser therapy” OR “light therapy” OR “low level laser” R “low intensity laser” OR “low power laser” OR “soft laser” OR “photo therapy OR photobiomodulation OR photostimulation) #3 TITLE-ABS-KEY (reten* OR relaps* OR retain* OR stab* OR mainten* OR reopen OR recur*) #4 #1 AND #2 AND #3 #5 #4 AND (LIMIT-TO (SUBJAREA, “DENT”)
PubMed	#1 orthodontics (Mesh) OR “orthodontic force” OR “active force” OR “appliance” #2 recurrence (Mesh) OR retention OR stability OR maintenance OR relapse OR “post ortho*” OR “remov* force #3 laser therapy (Mesh) OR “light therapy” OR “laser irradiation” OR phototherapy OR photobiomodulation OR “diode laser” OR “low power laser” OR “low intensity laser” OR “soft laser” OR “therapeutic laser” #4 #1 AND #2 AND #3
ProQuest	ab(retention OR relapse OR stability OR maintenance OR retainer OR reopen OR recurrence) AND ab(orthodontic) AND ab(laser therapy OR light therapy OR laser irradiation OR phototherapy OR photobiomodulation OR diode laser OR low power laser OR low intensity laser OR soft laser OR therapeutic laser)
